# Diagnostic Performance of GeneXpert BC as a Triage Test for Patients Presenting with Macroscopic Hematuria Suspicious for Urinary Bladder Cancer: A Multicenter Prospective Case-Control Study

**DOI:** 10.1016/j.euros.2024.10.016

**Published:** 2024-11-02

**Authors:** Suleiman Abuhasanein, Jonatan Radmann, Staffan Jahnson, Henrik Kjölhede

**Affiliations:** a Department of Urology, Institute of Clinical Science, Sahlgrenska Academy, University of Gothenburg, Göteborg, Sweden; b Department of Surgery, Urology Section, NU Hospital Group, Region Västra Götaland Health System, Uddevalla, Sweden; c Department of Urology, Varberg/Halmstad Hospital, Region Halland Health System, Halmstad, Sweden; d Department of Clinical and Experimental Medicine, Division of Urology, Linköping University, Linköping, Sweden; e Department of Urology, Sahlgrenska University Hospital, Göteborg, Sweden

**Keywords:** Bladder cancer, Diagnostic accuracy, Early detection, GeneXpert, Hematuria, mRNA

## Abstract

**Background and objective:**

Our objective was to assess whether GeneXpert BC can be used as a triage test to exclude urinary bladder cancer (UBC) for patients with macroscopic hematuria.

**Methods:**

We conducted a prospective study that include consecutive patients being evaluated for macroscopic hematuria between September 2020 and December 2022. Before cystoscopy, study participants provided a voided urine sample for GeneXpert BC analysis according to a case-control design with an emphasis on UBC detection. Descriptive statistics are reported for patient and tumor characteristics. To assess the diagnostic accuracy of the GeneXpert BC test, the sensitivity, specificity, and negative predictive value (NPV) were calculated, using the histopathologically proven UBC as the ground truth.

**Key findings and limitations:**

In total, 1505 subjects presenting with macroscopic hematuria were enrolled in the study. After randomization and exclusions, GeneXpert BC testing was carried out for 312 participants. Of these, 151 patients from the case arm had UBC, 122 patients from the control arm (random 10%) were negative for UBC, and 39 patients from the case arm did not have malignancy. Using a predefined linear discriminant analysis (LDA) threshold of ≥0.22, the test had sensitivity of 0.94 (95% confidence interval [CI] 0.90–0.97), specificity of 0.52 (95% CI 0.42–0.59), and NPV of 0.99 (95% CI 0.98–0.99). All false-negative tumors were of low grade (Ta grade 1–2). Cystoscopy and computed tomography urography could have been omitted in 44% of the patients with macroscopic hematuria. At a secondary LDA threshold of ≥0.45, the test had sensitivity of 0.79 (95% CI 0.73–0.86), specificity of 0.83 (95% CI 0.76–0.89), and NPV of 0.97 (95% CI 0.96–0.98).

**Conclusions and clinical implications:**

GeneXpert BC is a reliable triage test for deciding on whether further investigations are necessary in patients with macroscopic hematuria.

**Patient summary:**

We assessed a test called GeneXpert BC for the detection of bladder cancer in patients with blood in their urine. GeneXpert BC performed well in ruling out bladder cancer for patients who did not have cancer according to further tests. Use of GeneXpert BC could help in avoiding scans and invasive tests for patients with a negative result.

## Take Home Message

Results from our prospective study demonstrate that GeneXpert BC could potentially serve as an accurate and reliable triage test for patients with macroscopic hematuria and could help in avoiding further investigations in a substantial number of patients.

## 1 Introduction

Urinary bladder cancer (UBC) , which frequently presents with macroscopic hematuria, is among the most prevalent malignancies globally [1], [2]. Evaluation for patients with suspected UBC comprises cystoscopy and computed tomography urography (CTU) [1]. In a comprehensive systematic review comprising 229 701 participants, the pooled incidence rate for UBC in the cohort of patients with macroscopic hematuria was 17% (95% confidence interval [CI] 14–20%) [3]. Thus, while macroscopic hematuria is a strong indicator of UBC [4], up to 75–90% of patients with this sign do not have UBC. Nevertheless, patients with macroscopic hematuria still undergo both cystoscopy and CTU to exclude UBC and other more infrequent urological malignancies [5].

Both cystoscopy and CTU are invasive procedures, and investigation for large numbers of patients can be difficult to organize, which might result in undesirable delays in diagnosis [6]. Cystoscopy can cause significant patient discomfort, carries a risk of infection, and may fail to detect high-grade tumors [7], [8]. Similarly, CTU exposes patients to radiation, and injection of contrast medium increases the risk of secondary cancers and nephropathy [9], [10]. Finally, the financial health care burden of unnecessary cystoscopy and CTU should not be underestimated [11]. In a mixed-methods study investigating patient viewpoints on the acceptability of a urinary biomarker test as a substitute for cystoscopy, approximately four out of five participants expressed a willingness to accept a urine biomarker provided its sensitivity matched that of cystoscopy, and the majority would not accept a biomarker with a sensitivity lower than 0.90 [12].

GeneXpert BC is a new noninvasive assay [13], [14], but data on its diagnostic accuracy are still too immature to allow wide implementation as a triage test for patients with macroscopic hematuria in the clinical setting. Therefore, the objective of this study was to assess the diagnostic accuracy of GeneXpert BC in a general population of patients with macroscopic hematuria and whether it could be used as a triage test to exclude UBC and thereby omit further evaluation. The primary aim was to assess the diagnostic performance and clinical implications at a prespecified diagnostic threshold optimized to minimize false negatives. Secondary aims were to assess the diagnostic performance at the default threshold, and to calculate the proportion of patients for whom cystoscopy and CTU could be avoided.

## 2 Patients and methods

The study was designed as a randomized case-control diagnostic study [15]. This design was selected as it allows substantial reductions in costs and efforts associated with data collection and analysis in comparison to a full-cohort approach, while still offering a robust estimation of all accuracy parameters [16]. The study was registered on ISRCTN (ISRCTN17940603) before initiation.

### 2.1 Study subjects

The study involved prospective inclusion of consecutive patients being evaluated for macroscopic hematuria at three participating centers in Sweden (NU Hospital Group, Hallands Hospital, and Sahlgrenska University Hospital). Inclusion started on September 7, 2020 and ended on December 31, 2022. The inclusion criteria were referral for at least one episode of macroscopic hematuria (with or without other symptoms), age >50 yr, and ability to provide signed informed consent. Patients with a history of previous UBC or upper tract urothelial carcinoma (UTUC) were excluded, as were patients with an indwelling catheter and those unable to void, as the assay is only valid for voided urine samples. The criteria for a valid urine sample are described in the next section.

### 2.2 Study-specific collection and analyses

Typically, almost all patients underwent CTU performed and evaluated by an experienced radiologist before cystoscopy. Immediately before cystoscopy, participants provided a voided urine sample and filled out a questionnaire regarding symptoms and UBC risk factors. Patients were divided into a case arm, in which all patients had suspicion of UBC or UTUC according to cystoscopic or radiologic signs, and a control arm, in which patients primarily tested negative for UBC or UTUC. All urine samples in the case arm and a randomized selection (1:9) from the control arm were analyzed using the GeneXpert BC assay (Cepheid, Sunnyvale, CA, USA). We used a stratified randomized block design by drawing from a box at each center, with blocks of 100 assignments generated as needed.

Within 30 min of urine collection, a 4.5-ml aliquot was mixed with the GeneXpert BC preservative and stored at room temperature. The sample was then analyzed within 7 d using a GeneXpert BC detection cartridge on the Cepheid GeneXpert multimodule system and the total linear discriminant analysis (LDA) score was reported. LDA assigns each test result a numerical score according to the combination of measured variables that best discriminates between two classes or groups. By setting threshold values for these scores, results can be classified as either positive or negative for the condition being tested (UBC in our study). The threshold value is often determined via statistical analysis and validation studies to optimize the diagnostic accuracy and clinical utility. A primary threshold of LDA ≥ 0.22 was chosen on the basis of a previous study [14] and unpublished results (Cepheid, personal communication, June 2020), which represents estimated sensitivity of 0.90–0.95 and specificity of approximately 0.50. A secondary threshold of LDA > 0.45, which is the standard cutoff for the test, was also evaluated.

GeneXpert BC measures five mRNA markers *ABL1*, *UPK1B*, *IGF2*, *CRH*, and *ANXA10*. *ABL1* serves as a reference to verify the presence of human RNA in the urine sample [13]. Consequently, a urine specimen was deemed valid if it originated from voided urine, was positive for *ABL1*, and had a total LDA score falling within the valid range of −20 to 20. The clinical investigators and patients were blinded to the assay results. All patients in the case arm underwent transurethral resection of bladder tumor (TURBT) for histopathological verification of UBC, or biopsy or radical surgery for UTUC. We conducted 12-mo longitudinal follow-up of medical records for all subjects who initially tested negative for UBC or UTUC to detect any subsequent diagnoses of UBC or UTUC.

### 2.3 Statistical analysis

A sample size calculation for which sensitivity of 0.95 was anticipated indicated that 155 subjects with UBC would be needed to show sensitivity better than ∼0.90 with a one-sided α level of 0.05 and β level of 0.80. To allow for cases with unclear findings at cystoscopy and an expected UBC/UTUC prevalence of ∼8%, a total of about 2000 subjects was planned for inclusion, with inclusion to be halted after reaching the target number of UBC patients. This expected prevalence, which is lower than what has previously been reported in the literature, was based on local data during the 2 yr before the study. In addition, national data for the Swedish standardized care pathway for macroscopic hematuria has had a near-constant UBC/UTUC rate of approximately 10% since its inception in 2015.

Descriptive statistics are reported for patient and tumor characteristics. Continuous data are presented as the median with interquartile range (IQR). To assess the diagnostic accuracy of GeneXpert BC, the sensitivity, specificity, negative predictive value (NPV), and positive predictive value were calculated on the basis of histopathologically proven UBC or UTUC within 1 yr of enrolment. These values were calculated using inverse probability weighting according to the probability of the subject’s urine sample being analyzed according to the cystoscopy and CTU results.

Subgroup analyses for subjects with low-grade UBC (Ta grade 1–2) versus high-grade UBC (Ta grade 3, carcinoma in situ, T1–4), male versus female patients, smokers (including former smokers) versus never-smokers, and different age groups were also performed. In addition, receiver operating characteristic (ROC) curves were plotted and the area under the curve (AUC)with 95% CI was calculated for all UBC patients and for those with low-grade versus high-grade disease separately. To account for the sampling of control subjects and patients in the case arm for whom no malignancy was confirmed, inverse probability weighting was used in plotting the ROC curves and calculating the AUC. The proportion of patients who could avoid further evaluation with cystoscopy and CTU was calculated for both the primary and secondary thresholds. All CIs in the study were calculated by bootstrapping 1000 times, using inverse probability weighting, and taking the 2.5 and 97.5 percentiles as the lower and upper bounds, respectively [17]. Statistical analysis was performed using SPSS version 29 (IBM Corp., Armonk, NY, USA) and R version 4.3.3 (R Foundation for Statistical Computing, Vienna, Austria).

## 3 Results

**Fig. 1:**
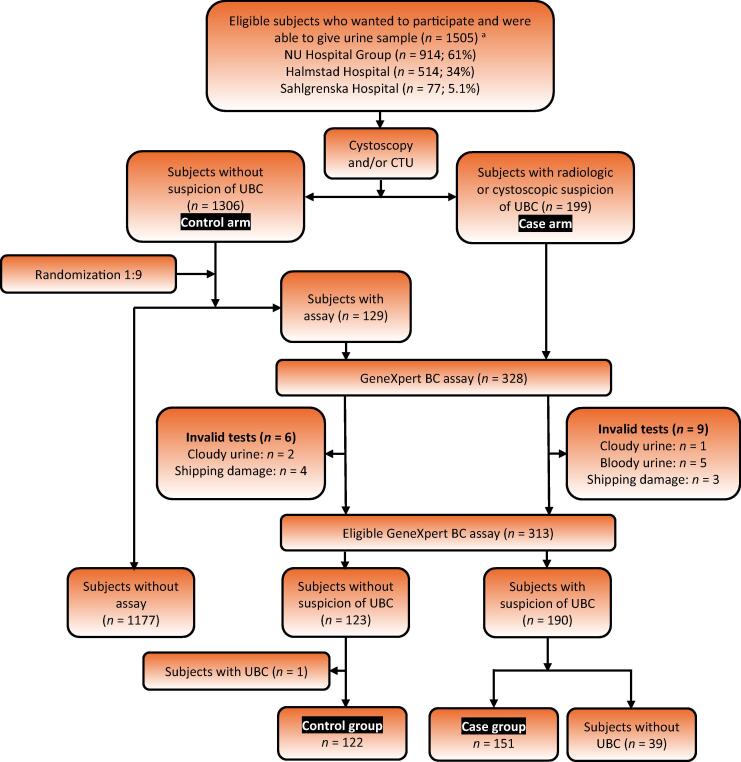
Flowchart of patient inclusion in the study. CTU = computed tomography urography; UBC = urinary bladder cancer. ^a^ Inclusion was between September 2020 and December 2022 in NU Hospital Group, between June 2021 and December 2022 in Halmstad Hospital, and between June 2022 and December 2022 in Sahlgrenska Hospital.

A group of 1505 subjects presenting with macroscopic hematuria was enrolled from September 2020 to December 2022 in the three participating centers (Fig. 1). A total of 199 subjects (13%) had cystoscopic or radiologic suspicion of UBC or UTUC and were included in the case arm. Of the remaining 1306 subjects (87%) in the control arm, 129 were randomly assigned to analysis with the GeneXpert BC assay. In the analytical cohort of 328 urine assays (199 case + 129 control), 15 samples (4.6%) had invalid results and were excluded from the analysis. One patient in the control group who was later found to have UBC was also excluded from the analysis. This resulted in a total of 312 subjects in the final study cohort: a case group of 151 patients who had UBC or UTUC, an additional 39 patients from the case arm who did not have malignancy, and a control group of 122 patients who did not have UBC or UTUC. Details for the study population are summarized in Table 1.

**Table 1:** Characteristics of the study population

Parameter	Case arm		Control arm	
	Included in final analysis	UBC absent	Included in final analysis)	No assay
Patients, *n* (%) a	151 (10)	39 (2.6)	122 (8.2)	1177 (78)
Male, *n* (%)	112 (74)	27 (69)	70 (57)	600 (51)
Median age, yr (IQR)	74 (66–80)	75 (67–81)	69 (61–75)	69 (60–76)
Hospital, *n* (%)				
NU Hospital Group	79 (52)	17 (44)	73 (60)	731 (62)
Hallands Hospital	65 (43)	17 (44)	45 (37)	386 (33)
Sahlgrenska Hospital	7 (4.6)	5 (13)	4 (3.3)	60 (5.1)
Smoking history, *n* (%)				
Never smoker	45 (31)	14 (36)	51 (42)	547 (46)
Former smoker	78 (53)	18 (46)	59 (49)	546 (46)
Current smoker	24 (16)	7 (18)	11 (9.0)	84 (7.1)
Median smoking duration, yr (IQR) b	29 (15–40)	27 (11–50)	17 (10–30)	20 (10–30)
History of snuff use, *n* (%)				
Never user	116 (79)	31 (80)	103 (85)	939 (80)
Former user	7 (4.6)	2 (5.1)	7 (5.7)	121 (10)
Current user	24 (16)	6 (15)	11 (9.0)	117 (10)
Median duration of snuff use, yr (IQR) b	20 (8–40)	15 (5–37)	20 (10–30)	20 (7–34)
Urgency, *n* (%)	48 (33)	15 (39)	53 (44)	536 (45)
Dysuria, *n* (%)	33 (22)	12 (31)	42 (43)	496 (42)
Lower abdominal pain, *n* (%)	26 (18)	8 (21)	30 (25)	324 (27)
Visual urine appearance, *n* (%)				
Clear	114 (76)	31 (79)	113 (93)	1093 (93)
Bloody	31 (20)	5 (13)	6 (4.9)	39 (3.3)
Cloudy	6 (4.0)	3 (7.7)	3 (2.5)	45 (3.8)
Antithrombotic medication, *n* (%)	45 (30)	16 (41)	31 (25)	365 (31)
Median LDA score (IQR)	0.91 (0.59–1.21)	0.45 (0.24–0.57)	0.21 (0.11–0.36)	NA

LDA = linear discriminant analysis; IQR = interquartile range; UBC = urinary bladder cancer.

a Not including the 15 patients with invalid tests and one patient with a false-negative cystoscopy result.

b For both former and current users.

**Fig. 2:**
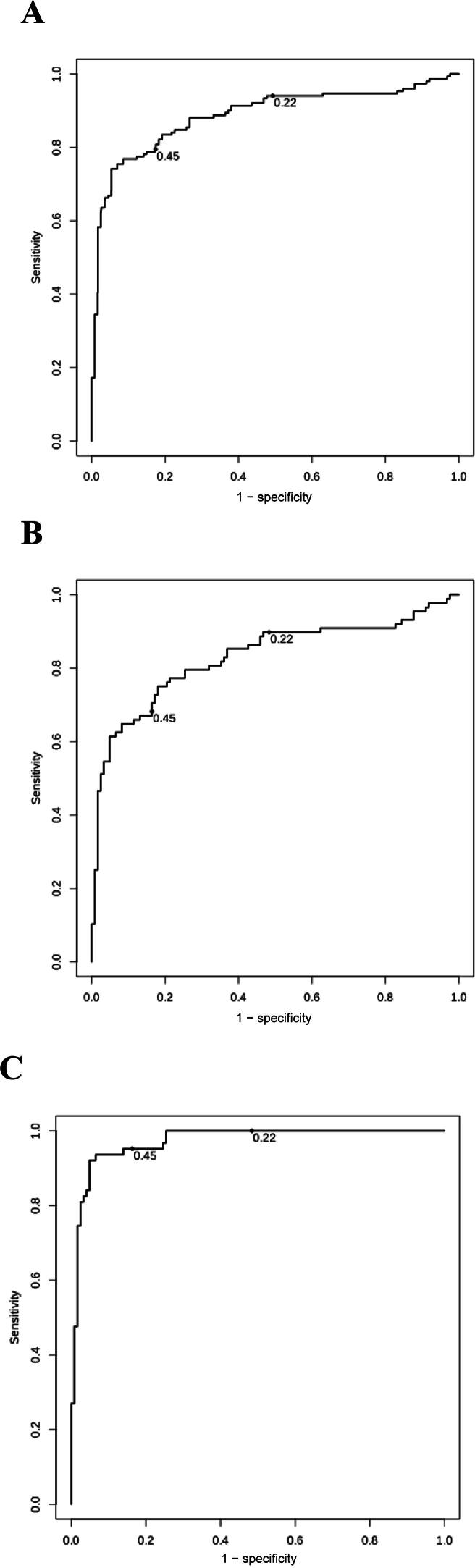
Receiver operating characteristic curves for the GeneXpert BC after 1 yr of follow-up and transurethral resection of bladder tumor for suspected UBC cases. (A) All patients included in the study (AUC 0.89, 95% CI 0.85–0.93). (B) All patients with low-grade UBC included in the study (AUC 0.83, 95% CI 0.77–0.89). (C) All patients with high-grade UBC included in the study (AUC 0.97, 95% CI 0.95-0.99). Points at 0.22 and 0.45 in the plots show the threshold points chose. AUC = area under the receiver operating characteristic curve; CI = confidence interval; UBC = urinary bladder cancer.

The median LDA score from the GeneXpert BC assay was 0.21 (IQR 0.11–0.36) in the control group and 0.91 (IQR 0.59–1.21) in the case group. For patients in the case arm who did not have a confirmed malignancy (*n* = 39) the median LDA was 0.45 (IQR 0.24–0.57). ROC curve analysis showed an overall AUC of 0.89 (95% CI 0.85–0.93; Fig. 2). Subgroup analyses showed AUCs of 0.83 (95% CI 0.77–0.89) for low-grade UBC and 0.97 (95% CI 95–99%) for high-grade UBC.

At the predefined primary threshold of LDA ≥ 0.22, there were nine cases with a false-negative result and 90 with a false-positive result, giving weighted sensitivity of 0.94 (95% CI 0.90–0.97), specificity of 0.52 (95% CI 0.42–0.59), and NPV of 0.99 (95% CI 0.98–0.99) (Table 2). The sensitivity was 0.90 (95% CI 0.83–0.95) or low-grade disease and 1.0 (95% CI 1.0–1.0) for high-grade disease. All nine patients with false-negative results (6.0% of all UBC/UTUC cases) had only low-grade UBC (Ta grade 1–2). For patients with no other symptoms than hematuria, the sensitivity and specificity were similar. When using the secondary threshold (LDA ≥ 0.45), all patients with UTUC were also identified. At this threshold the overall sensitivity was 0.79 (95% CI 0.73–0.86), overall specificity was 0.83 (95% CI 0.76–0.89), and overall NPV was 0.97 (95% CI 0.96–0.98). The results are listed in Table 3.

**Table 2:** Performance metrics for the GeneXpert BC assay stratified by demographic factors, tumor grade, and LDA threshold a

Variable	TP	TN	FP	FN	Prev.	Sensitivity (95% CI)	Specificity (95% CI)	PPV (95% CI)	NPV (95% CI)
**LDA threshold ≥0.22**									
All patients	142	71	90	9	0.10	0.94 (0.90–0.97)	0.51 (0.42–0.59)	0.18 (0.15–0.22)	0.99 (0.98–0.99)
Sex									
Male	104	43	54	8	0.13	0.93 (0.88–0.97)	0.51 (0.40–0.62)	0.22 (0.18–0.27)	0.98 (0.96–0.99)
Female	38	28	36	1	0.07	0.97 (0.92–1.00)	0.51 (0.38–0.65)	0.12 (0.10–0.18)	1.0 (0.99–1.0)
Age									
<60 yr	17	12	17	0	0.06	1.0 (1.0–1.0)	0.42 (0.24–0.61)	0.10 (0.07–0.14)	1.0 (1.0–1.0)
60–70 yr	33	22	28	2	0.08	0.94 (0.86–1.0)	0.48 (0.33–0.64)	0.13 (0.10–0.19)	0.99 (0.97–1.0)
≥70 yr	92	37	45	7	0.14	0.93 (0.87–0.97)	0.56 (0.44–0.69)	0.26 (0.20–0.33)	0.98 (0.96–0.99)
Smoking status									
C/F smoker	95	43	52	7	0.12	0.93 (0.88–0.98)	0.53 (0.41–0.64)	0.21 (0.17–0.27)	0.98 (0.97–0.99)
Never smoker	43	27	38	2	0.08	0.96 (0.89–1.0)	0.46 (0.34–0.60)	0.13 (0.10–0.17)	0.99 (0.98–1.0)
Other symptoms									
No	71	29	31	4	0.15	0.95 (0.89–0.99)	0.55 (0.40–0.70)	0.26 (0.21–0.35)	0.98 (0.96–1.0)
Yes	67	41	59	5	0.08	0.93 (0.87–0.99)	0.48 (0.38–0.58)	0.13 (0.11–0.17)	0.99 (0.98–1.0)
Malignancy									
Low grade	79	63	59	9	0.07 b	0.90 (0.83–0.95)	0.52 (0.43–0.61)	0.12 (0.10–0.14)	0.99 (0.98–0.99)
High grade	63	63	59	0	0.05 c	1.0 (1.0–1.0)	0.52 (0.43–0.61)	0.09 (0.08–0.12)	1.0 (1.0–1.0)
**LDA threshold ≥0.45**									
All patients	120	122	39	31	0.10	0.79 (0.73–0.86)	0.83 (0.76–0.89)	0.35 (0.27–0.47)	0.97 (0.96–0.98)

C/F = current/former; CI = confidence interval; LDA = linear discriminant analysis; FN = false negative; FP = false positive; NPV = negative predictive value; PPV = positive predictive value; Prev. = prevalence; TN = true negative; TP = true positive.

a Accuracy parameters were calculated using inverse probability weighting and CIs were calculated via bootstrapping.

b Excluding patients with high-grade cancer.

c Excluding patients with low-grade cancer.

**Table 3:** Characteristics of patients with UBC or UTUC grouped by GeneXpert BC assay result

Variable	LDA< 0.22	0.22 ≤ LDA < 0.45	LDA ≥ 0.45
Patients, *n* (%)	90 (28)	73 (23)	159 (49)
UBC cases, *n* (%)	9 (6.0)	22 (15)	120 (79)
UTUC cases, *n* (%)	0	0	6 (100)
**UBC group**			
Male, *n*/145 (%)	8 (89)	15 (68)	89 (74)
Median age, yr (IQR)	76 (74-77)	70 (61-76)	74 (67-80)
Solitary tumor, *n*/145 (%) a	7 (78)	18 (82)	72 (60)
Tumor size <3 cm, *n*/135 (%) a	8 (100)	16 (89)	84 (70)
Tumor stage and grade, *n*/145 (%) b			
R cTa grade 1–2	9 (100)	19 (86)	59 (49)
R cTa grade 3, Tis, T1	0	2 (9.1)	47 (39)
R ≥cT2	0	1 (4.5)	14 (12)
**UTUC group**^c^			
Male, *n*/6 (%)			3 (50)
Median age, yr (IQR			73 (65–78)
Tumor site, *n*/6 (%)			
Renal pelvis			2 (33)
Ureter			4 (67)
Tumor stage and grade, *n*/6 (%)			
cTa grade 1–2			1 (16)
cTa grade 3, Tis, T1			2 (34)
≥cT2			3 (50)

LDA = linear discriminant analysis; IQR = interquartile range; UBC = urinary bladder cancer; UTUC = upper tract urothelial cancer

a According to transurethral resection of bladder tumor.

b Corrected after second-look resection if appropriate.

c There were no cases with UTUC and a negative GeneXpert BC assay.

Using inverse probability weighting (probability for cases = 1; probability for suspected cases without UBC/UTUC = 1; probability for controls = 128/1305) we found that 60.3 (4.0%) would have invalid assays, 778.8 (52%) would have a positive test, and 654.9 (44%) would have a negative test using the primary threshold. Bootstrapping showed a 95% CI of 36–52% for the proportion of patients who could potentially have avoided cystoscopy and CTU. There were no subgroups for which the proportion of avoidable cystoscopies was significantly different in a clinically meaningful sense. For the secondary threshold (LDA ≥ 0.45), 73% (95% CI 67–79%) of patients could potentially have avoided further evaluation. The number of cystoscopies needed to detect one case (number needed to examine) with Ta grade 1–2 tumor with a false-negative assay would therefore have been 654.9/9 = 72.8 (95% CI 59.8–86.0) at the primary threshold. For the secondary threshold, 1098.3/31 = 35.4 (95% CI 32.1–38.1) cystoscopies would be needed to detect one further case of UBC.

## 4 Discussion

Our results demonstrate the efficiency of GeneXpert BC as a reliable triage test for assessment of macroscopic hematuria, achieving sensitivity of 0.94 and NPV of 0.99. This finding is consistent with results reported for other studies [14], [18], [19]. In a study involving 156 patients with either microscopic or macroscopic hematuria, Kavcic et al [18] found sensitivity and NPV of 1.00. Our study had a significantly larger population and focused on individuals with macroscopic hematuria, who represent a cohort more susceptible to UBC/UTUC than those with only microscopic hematuria. In our study, the AUC of 0.87 is similar to the value of 0.86 reported by Kavcic et al [18], indicating robust performance of GeneXpert BC.

Elsawy et al [19] conducted a study involving 181 patients with hematuria and reported GeneXpert BC sensitivity of 0.74 (95% CI 0.67–0.79) and NPV of 0.92 (95% CI: 0.89–0.96). For high-grade UBC, Elsawy et al reported NPV of 1.00, which is the same as in our study. The lower sensitivity observed in their study can probably be attributed to the smaller number of cancer cases they included (36 cases). A multicenter study by Wallace et al [14] revealed sensitivity of 0.73 and specificity of 0.90 at an LDA threshold of 0.40 in a cohort of 93 patients presenting with macroscopic hematuria. By contrast, we used a lower threshold and observed higher sensitivity and lower specificity. We used a lower threshold to prioritize high sensitivity, as our primary goal was to establish a method for excluding UBC, which is more clinically relevant as a triage test.

We demonstrated that approximately 44% of cystoscopies could be omitted for primary evaluation of macroscopic hematuria, in accordance with Hurle et al [20], who showed that GeneXpert BC use in practice could reduce the need for cystoscopy by 34%. Nonetheless, it is important to note that we applied a lower threshold (LDA ≥ 0.22) than Hurle et al [20] (LDA ≥ 0.40), which explains the low sensitivity reported in this study. In a study by D’Elia et al [21] involving patients undergoing ureteropyeloscopy for suspected UTUC, the sensitivity was 0.83 and NPV was 0.90 using voided urine and a high threshold of LDA ≥ 0.45. This eliminated the need for unnecessary ureteropyeloscopy and reduced the associated risks and complications. These results are in line with our findings that no patient with UTUC had LDA <0.45.

CTU has limited sensitivity for the detection of small lesions in the upper urinary tract; therefore, urologists sometimes rely on urine cytology as an adjunctive test [22]. However, urine cytology faces challenges such as limited sensitivity for low-grade UBC (0.16) [23], variability in cytopathology interpretation, and potential sampling errors during collection [24]. We did not include cytology in the research protocol as it is not used in our routine practice. Cystoscopy also has limitations as it does not guarantee 100% accuracy, with sensitivity ranging from 0.87 to 1.00, and NPV from 0.98 and 1.00 [25]. There is a clear clinical need for new noninvasive diagnostic tests for better patient selection for cystoscopy and thereby reduce unnecessary testing and costs. The low risk of missing high-grade UBC or UTUC with either threshold in the present study suggests that it is feasible to avoid these investigations in a large proportion of patients. However, cystoscopy might still be performed for other reasons, such as persistent or recurrent macroscopic hematuria.

A smaller proportion of patients in our study had UBC or UTUC (10%) in comparison to previous studies [4], [26]. This difference can be attributed to the exclusion of some elderly patients, who represent a population with high UBC prevalence. These individuals were excluded because they were unable to provide informed consent. Furthermore, we excluded patients with an indwelling catheter who encountered bleeding possibly caused by a large UBC tumor. Cancer markers are generally more accurate for larger or multiple UBC tumors and in cases of advanced disease, suggesting that identification of smaller and less aggressive tumors is more challenging [27]. However, in our cohort characterized by smaller and less aggressive tumors, GeneXpert BC successfully detected the majority of the tumors. It is possible that the number of avoidable cystoscopies would be different in other clinical settings with higher UBC prevalence. It would therefore be valuable to repeat the study in non-Nordic countries.

Besides new liquid-biopsy triage tests for urothelial cancer, the entire diagnostic pathway could be further improved by new technology. To mitigate the risks associated with TURBT, a noninvasive approach for local staging of UBC would offer significant benefits. A study on the use of micro-ultrasound showed a 29% increase in upstaging [28]. However, micro-ultrasound is not without limitations and the evidence is still limited. Integration of artificial intelligence in bladder cancer diagnostics has also emerged as a promising avenue in terms of early detection and precise diagnosis [29].

Our study has several strengths. First, it is a prospective multicenter study with a substantial number of patients, which enhances its statistical power and minimizes selection bias. Furthermore, we only included patients with macroscopic hematuria, a cohort with higher risk of UBC, whereas other studies included both microscopic and macroscopic hematuria cases. Moreover, we did not exclude patients with a history of urinary stone disease, ongoing urinary tract infection, anticoagulant use, or a recent endoscopic procedure in the urinary tract in an effort to mirror the real-world scenario for the population with macroscopic hematuria.

Our study also has some limitations. Although case-control studies can be cost effective in comparison to full-cohort designs, potential for selection bias arises if the control group selected does not accurately mirror the cohort from which the cases emerge. To address this concern, we used randomization for selection of control subjects. Since sensitivity was the primary focus in our study, the emphasis was on maximizing the number of cases according to the resources available. Furthermore, cystoscopy and CTU are imperfect diagnostic tools, leading to initial inclusion of 39 patients for whom UBC was subsequently not confirmed, which affected the statistical analysis. The inference for the number of cystoscopy procedures that could be avoided may have been influenced, and this will need to be tested in a full-cohort study. In addition, we did not evaluate whether other concomitant symptoms would lead to cystoscopy, which could affect the conclusions regarding avoidable procedures.

Another limitation associated with mRNA-based markers is the challenge of obtaining an adequate quantity of high-quality RNA from voided urine samples. To address this issue, a positive ABL1 signal, confirming the presence of sufficient human RNA in the urine sample, is required for the test to be considered valid. In our study, only a small fraction of test results were invalid. A further limitation is the underutilization of cytology in the study. However, there were no missed tumors that would have been detected via cytology. Moreover, the aim of our study was to determine whether GeneXpert BC could be used as an isolated triage test to exclude UBC, for which cytology would not be useful. Lastly, we did not evaluate how many cases of renal cell carcinoma would have been missed by omitting CTU in a large proportion of patients. The number of clinically significant kidney tumors that actually caused macroscopic hematuria is likely to be very low and these cases would probably be detected at repeat episodes of macroscopic hematuria. The question of whether opportunistic screening for small kidney tumors in this population is of clinical value might arise. However, this should be evaluated in further full-scale clinical trials.

## 5 Conclusions

GeneXpert BC is an effective triage test in the diagnostic setting for patients with macroscopic hematuria and can help in safely avoiding cystoscopy and CTU in almost half of cases. Thereby leading to a faster and more effective process for evaluating macroscopic hematuria. Further comprehensive clinical trials are warranted.

  ***Author contributions***: Suleiman Abuhasanein had full access to all the data in the study and takes responsibility for the integrity of the data and the accuracy of the data analysis.

  *Study concept and design*: Abuhasanein, Kjölhede.

*Acquisition of data*: Abuhasanein, Radmann.

*Analysis and interpretation of data*: Abuhasanein, Jahnson, Kjölhede.

*Drafting of the manuscript*: Abuhasanein, Kjölhede.

*Critical revision of the manuscript for important intellectual content*: Abuhasanein, Radmann, Jahnson, Kjölhede.

*Statistical analysis*: Abuhasanein, Kjölhede.

*Obtaining funding*: Abuhasanein, Kjölhede.

*Administrative, technical, or material support*: Abuhasanein, Kjölhede.

*Supervision*: Abuhasanein, Kjölhede.

*Other*: None.

  ***Financial disclosures:*** Suleiman Abuhasanein certifies that all conflicts of interest, including specific financial interests and relationships and affiliations relevant to the subject matter or materials discussed in the manuscript (eg, employment/affiliation, grants or funding, consultancies, honoraria, stock ownership or options, expert testimony, royalties, or patents filed, received, or pending), are the following: None.

  ***Funding/Support and role of the sponsor*:** This study was supported by grants from the Swedish state under an ALF agreement between the Swedish government and the county councils (ALFGBG-873181), the Swedish Society of Medicine (SLS-890771), and the Department of Research and Development, NU Hospital Group. The sponsors played no direct role in the study. Cepheid supplied the GeneXpert BC analysis kits at a discount, but the company had no involvement in the study process.

  ***Acknowledgments***: We thank Rajaa Hassan for technical support and Anna Levinsson and Erik Bülow for statistical support. We also thank all personnel in the Department of Laboratory Medicine of Västra Götalandsregionen and the Department of Microbiology, Hallands Hospital for their technical support. We are grateful to all staff members at the urology outpatient clinics in the NU Hospital Group, Halmstad/Varberg Hospital, and Sahlgrenska Hospital for their technical support and assistance in conducting the study.

  ***Ethics considerations***: The study was approved by the Swedish Ethical Review Authority (2019-05582) with an approved amendment (2021-01786) for addition of participating centers and was performed in accordance with good clinical practice guidelines and the Declaration of Helsinki.

  ***Data sharing statement***: The data used to support the findings of this study are available from the corresponding author on reasonable request.
