# Supplementary material for: Diagnostic Performance of GeneXpert BC as a Triage Test for Patients Presenting with Macroscopic Hematuria Suspicious for Urinary Bladder Cancer: A Multicenter Prospective Case-Control Study
Source: Eur Urol Open Sci. 2024 Nov 2;70:158–66. doi: 10.1016/j.euros.2024.10.016 (PMC11567930; doi:10.1016/j.euros.2024.10.016)
Supplement: Supplementary Data 1 [file mmc1.docx]

**Description of calculation of accuracy parameters**

For cases and controls alike, each group was divided into “invalids” (those with invalid test results), “erroneous” (those erroneously labelled as cases or controls, after histopathological results and one year of follow-up), and “true” (those correctly classified as cases or controls).

**Inverse sampling probability** = (all controls in the study randomized to test / all controls included in the study)^-1^ *This parameter was kept constant for all subgroup analyses and across each bootstrapped set.*

**True positives** = true cases with test LDA ≥ cutoff

**False negatives** = true cases with test LDA < cutoff

**True negatives** = true controls with test LDA < cutoff × inverse sampling probability + erroneous cases with test LDA < cutoff

**False positives** = true controls with test LDA ≥ cutoff × inverse sampling probability + erroneous cases with test LDA ≥ cutoff

**Sensitivity** = True positives / (True positives + False negatives)

**Specificity** = True negatives / (True negatives + False positives)

**Positive predictive value** = True positives / (True positives + False positives)

**Negative predictive value** = True negatives / (True negatives + False negatives)

**Omittable evaluations** = (True negatives + False negatives) / (True negatives + False negatives + True positives + False positives + “invalid” cases + “invalid” controls × inverse sampling probability)

**Bootstrapping for confidence intervals**

The case arm and the control arm were each bootstrapped separately 1000 times, and the accuracy parameters were calculated according to the above description. The 2.5^th^ and 97.5^th^ percentiles for each of these parameters were used as confidence intervals. In addition, the **AUC** of the ROC was calculated for each bootstrapped set and the same percentiles were used as confidence interval.
